# Simultaneous Measurements of Dose and Microdosimetric Spectra in a Clinical Proton Beam Using a scCVD Diamond Membrane Microdosimeter

**DOI:** 10.3390/s21041314

**Published:** 2021-02-12

**Authors:** Oluwasayo Loto, Izabella Zahradnik, Amelia Maia Leite, Ludovic De Marzi, Dominique Tromson, Michal Pomorski

**Affiliations:** 1 Université Paris-Saclay, CEA, List, F-91120 Palaiseau, France; Izabella.Zahradnik@cea.fr (I.Z.); dominique.tromson@cea.fr (D.T.); 2 Institut Curie, Radiation Oncology Department, PSL Research University, Proton Therapy Centre, Centre Universitaire, 91898 Orsay, France; amelia.maialeite@curie.fr (A.M.L.); ludovic.demarzi@curie.fr (L.D.M.); 3 Institut Curie, PSL Research University, University Paris Saclay, LITO, Inserm, 91898 Orsay, France

**Keywords:** diamond, proton therapy, microdosimetry, radiation detectors, dosimeters, sensors

## Abstract

A single crystal chemical vapor deposition (scCVD) diamond membrane-based microdosimetric system was used to perform simultaneous measurements of dose profile and microdosimetric spectra with the Y1 proton passive scattering beamline of the Center of Proton Therapy, Institute Curie in Orsay, France. To qualify the performance of the set-up in clinical conditions of hadrontherapy, the dose, dose rate and energy loss pulse-height spectra in a diamond microdosimeter were recorded at multiple points along depth of a water-equivalent plastic phantom. The dose-mean lineal energy (${\bar{y}}_{D}$) values were computed from experimental data and compared to silicon on insulator (SOI) microdosimeter literature results. In addition, the measured dose profile, pulse height spectra and ${\bar{y}}_{D}$ values were benchmarked with a numerical simulation using TOPAS and Geant4 toolkits. These first clinical tests of a novel system confirm that diamond is a promising candidate for a tissue equivalent, radiation hard, high spatial resolution microdosimeter in beam quality assurance of proton therapy.

## 1. Introduction

The use of proton beams for the treatment of cancers has gained considerable interest in recent years. This is observable from the increase in the number of proton beam centers around the world. For instance, in Europe there were 25 more proton beam centers constructed between 2009 and 2019 [1]. This is a result of the comparative advantage proton therapy offers over conventional photon radiation therapy. The absorbed energy of a proton beam increases with depth, whereas that of photons decreases with depth, giving clinical proton therapy a higher conformal dose delivery and hence less damage to critical tissues in patients than obtainable in photon radiation therapy. Like every other radiation procedure, there are some concerns around the undesirable effects of proton therapy, including necrosis of the healthy tissue in the proximity of a treated tumor and cases of secondary cancers being induced. The occurrence of secondary effects depends on the radiation dose and volume, and the region irradiated [2]. Studies [3,4,5] have revealed the need for LET optimization to reduce any unwanted biological effects in proton therapy. The relative biological effectiveness (RBE) of radiation is defined as the ratio $D_{L}/D_{H}$, where $D_{H}$ is the absorbed dose of radiation H at which the probability of a given biological effect is equal to that at an absorbed dose $D_{L}$ of a reference radiation, L [6]. The RBE has been found to depend on the deposited physical dose, irradiated tissue and the quality of the beam which is measured by the linear energy transfer (LET). The RBE is an experimental quantity obtained from irradiation studies on living cells. A proton RBE value of 1.1 is usually used for clinical therapy with photon radiation used as a reference [7]. Though the RBE model that proposes a value of 1.1 has been widely accepted, studies have shown that this value varies with proton energies (i.e., along the depth dose profile), increasing significantly at the distal part of the Bragg Peak. Hence, an RBE weighted treatment plan could lead to optimized dose delivery to patients and fewer secondary effects. Microdosimetry has been used in the investigation of microdosimetric quantities of the radiation field. The probability density d(y), together with the dose-averaged lineal energy, ${\bar{y}}_{D}$, are physical quantities correlated with the biological effectiveness of the therapeutic beam obtainable from microdosimetry measurements. Tissue equivalent proportional counters (TEPC) are well established for clinical microdosimetry. However, solid state microdosimeters have been introduced in clinical dosimetry, as they offer higher spatial resolution, more robustness and easier integration than conventional TEPCs.

Most solid state microdosimeters are based on silicon and have been introduced as alternatives to TEPCs. Due to the non-tissue equivalent nature of silicon, corrections must be made for effective extraction of dose parameters [8]. Over the years, since the first publication on silicon microdosimeters, there have been continuous improvements in the design, structure and efficiency [9,10,11,12,13]. Due to the limitations of silicon-based microdosimeters, further developments in the portability and efficiency of the TEPCs are also being considered. Some compelling work is that of Conte [14] and Bianchi [15] on the use of sealed mini TEPC for proton beam therapy. Bianchi et al. observed similar microdosimetry spectra for mini TEPC and a ΔE−E silicon microdosimeters at linear energies higher than 8 (keV/μm ) with discrepancies observed at lower LET values.

Rollet [16] first reported the use of artificial diamond for microdosimeters. Diamond (Z = 6) offers advantages due to its radiation hardness and near tissue equivalence (Z = 7.5) for photon radiation, resulting in a less variable function for energy loss spectra conversion to water compared, for example, to silicon (Z = 14) [17]. In addition, some of the physical and electronic properties of diamond, such as its large-band gap, temperature stability, fast drift velocity and low capacitance, make it an interesting potential material for producing microdosimetric devices. Previous research [18,19] has shown that single crystal diamond detectors exhibit good charge collection efficiency and homogeneity in the micro-sensitive volumes, and can be used in measuring microdosimetric quantities in clinical beams. Using new device structures [20], improvements of the electric field geometry have also been proposed to maximize the charge collection efficiency and full potential of these detectors.

A diamond guard ring microdosimeter previously reported [21] by this group has shown the possibility of obtaining a sensor with full charge collection efficiency over a broad range of linear energy transfer of ions at various energies. The proof of concept has prompted the authors to pursue the integration of this detector to simultaneously measure the depth dose profile and the microdosimetric spectra of a degraded 230 MeV clinical beam at the Proton therapy Center, Orsay, France.

## 2. Materials and Methods

### 2.1. scCVD Diamond Membrane Guard Ring (GR) Prototype

**Figure 1:**
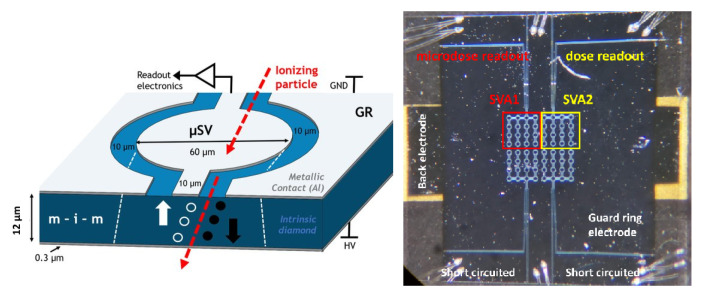
Left: the schematic representation of one microsensitive volume μSV of diamond GR microdosimeter prototype. Right: an optical micrograph of the diamond membrane with four sensistive microSVs arrays showing the μSVs marked with red (for microdosimetric read-out) and yellow (dose read-out) squares

The scCVD diamond membrane consists of four arrays of 16 sensitive volumes interconnected with bridges and bonded on a DIL20 chip carrier. Structurally, it comprises an intrinsic diamond layer (12 μm thickness) sandwiched between two layers of metallic aluminum electrodes. Further patterning of the top electrode and a chemical etching process were performed for the realization of multiple microsensitive volumes (μSVs) surrounded by the guard ring structure, as shown in Figure 1 (left). The critical dimensions have been chosen for the best signal to noise ratio in measurement conditions. Detailed descriptions of the fabrication process of a guard-ring (GR) scCVD membrane microdosimeter and its charge transport characterization with an ion microbeam can be found in previous work [21].

As shown in Figure 1 (right), the complete scCVD diamond membrane plate is arranged in four arrays hereafter referred to as Sensitive Volume Arrays (SVA). Each array comprises 16 μSVs interconnected with bridges and micro-bonded onto the chip carrier. In this device prototype, only two (SVA1 and SVA2) of the four arrays of SVAs were used—one for microdosimetric spectra acquisition and the other for dose measurements. The active area and lateral size of one SVA was 16 μSVs and 0.045 mm${}^{2}$, with each microsensitive volume having a 60 μm diameter. The total active area including bridges was 0.057 mm${}^{2}$. The thickness of the diamond membrane was 12 μm, as confirmed via alpha particle absorption spectroscopy and ion beam-induced current technique with the scanning transmission ion microscopy approach [20].

### 2.2. Measurement Set-Up

**Figure 2:**
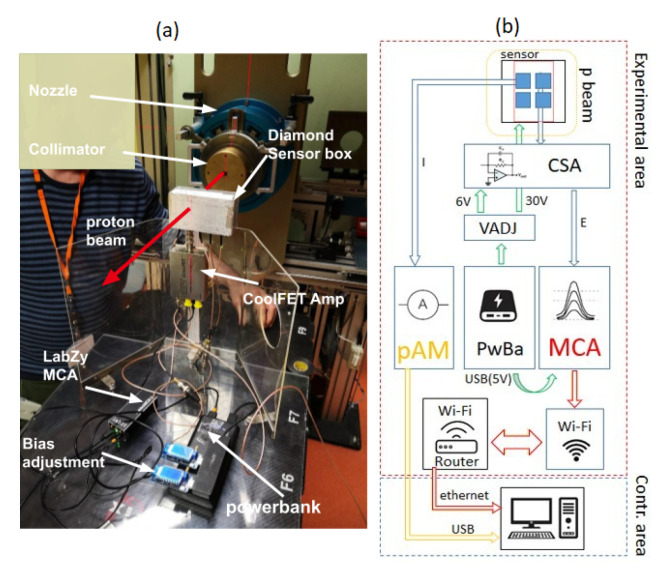
(**a**) A labelled photograph of the experimental set-up and (**b**) a schematic representation of the experimental set-up.

A battery powered, diamond-based dosimetric and microdosimetric measurement system has been developed. Although not fully integrated and miniaturized yet, here we present the first proof-of-principle of operation of the modular system in the clinical conditions of a hadrontherapy facility. Figure 2 (right) shows a diagram of the set-up used. The electrical signal induced by the proton beam in both SVA of the diamond sensor (enclosed in an electrically screened plastic housing) was fed to both a charge sensitive preamplifier Amptek CoolFET (CSA) for the purposes of microdosimetric spectra measurements and a high precision Keithley 6517 A pico-ammeter (pAM) for dose/dose rate measurement. A fixed bias voltage of 15 V (equivalent to electric field of 1.2 V/μm) was applied directly to the back electrode of the diamond sensor from a voltage adjustment element (VADJ). The system was powered by a 5 V, 24,000 mAh PowerBank (PwBa).

Pre-amplified voltage pulses from CSA induced in the SVA1 of the diamond sensor by single-particles are fed to a versatile, small multi-channel analyzer (LabZY nanoMCA II [22]) with an integrated digital amplifier and a WiFi module for data transfer. The multi-channel analyzer’s digital amplifier shapes and further amplifies the signal and processes the generated pulse-height spectra. Communication is done with the personal computer (PC) through a WiFi router. Physical connection between the PC (placed in control area) and the WiFi router (placed in experimental area) was accomplished by a 20 m long Ethernet cable. The beam-induced DC current from the SVA2 of the sensor was fed to the picoammeter (placed in the experimental) area through 2 m coaxial cable. Communication of the picoammeter with the PC, where data were stored, was assured through a 20 m USB cable with three repeaters.

### 2.3. Passive Scattering Beamline at the Proton Therapy Centre, Orsay

**Figure 3:**
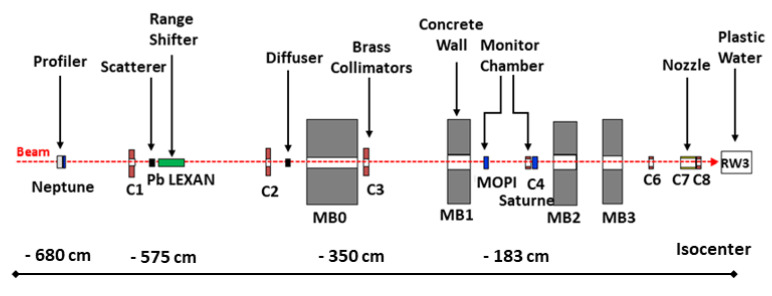
A schematic representation of the Y1 beam line of IC-CPO used in TOPAS/Geant4 simulation.

A proton beam of mean energy 230 MeV and energy spread 0.6 MeV (2σ) generated by an IBA C235 cyclotron, was degraded to 89 MeV in the isocenter by a range shifter. The range shifter contains a combination of lexan and lead layers of different thicknesses. The beam is further scattered by lead foils before entering the treatment room. A large brass collimator of 4×4 cm${}^{2}$ was placed at the end-point of the beam line, in order to obtain a homogenous 2D beam profile and at the same time protect readout electronics from radiation damage. The water equivalent phantom SP34 made of white polystyrene, type RW3 [23], in the form of square plates of various thicknesses, 10, 5 and 1 mm, were used to create dose profiles within target depth. These plates were gradually placed directly before sensor housing during irradiation. The uncertainty of positioning depth was estimated to be less than 0.1 mm, and it is mostly related to precision of plate fabrication and presence of air gaps between the plates. All the components of the experiment including the geometry of the beam line (shown in Figure 3) and the diamond sensor were included in obtaining the energy loss spectra in Geant4 and TOPAS numerical simulations. The Geant4 electromagnetic and hadronic physics models were used in the simulation for describing particle trajectories. Details of these models can be obtained in reference [24].

### 2.4. Energy Calibration Procedure for Microdosimetric Spectra Measurements

Calibration was done in the laboratory prior to the measurements at a hadron therapy facility. The procedure includes a combination of two techniques: First, the measurement of 5.486 MeV alpha particles spectra from Am-241 with 300 μm thick fully absorbing scCVD diamond detector in a vacuum and secondly, a consecutive pulse generator calibration. A thick electronic grade scCVD detector was placed in a vacuum chamber biased at 300 V (1 V/μm). Using an alpha particles radioactive source, the pulse height spectra was recorded. A precise line spectra was obtained and fitted with a gaussian distribution. The centroid at a fixed channel number corresponds to 5.486 MeV and at full width half maximum of the alpha line of 0.3%. Consequently an ORTEC pulse generator amplitude was adjusted to match perfectly the measured alpha spectra line. Then, the thick scCVD detector was replaced by the scCVD diamond membrane microdosimeter reported in this work. The system was re-calibrated with the pulse generator, introducing 5 spectral lines at 5.486, 2.743, 1.37, 0.685 and 0.343 MeV. No shift in the pulse generator peak at 5.486 MeV was observed compared to the measurement system with thick diamond detector, indicating negligible influence of different detector capacitance on the calibration process.

## 3. Results

### 3.1. Dosimetric Performance of the System

**Figure 4:**
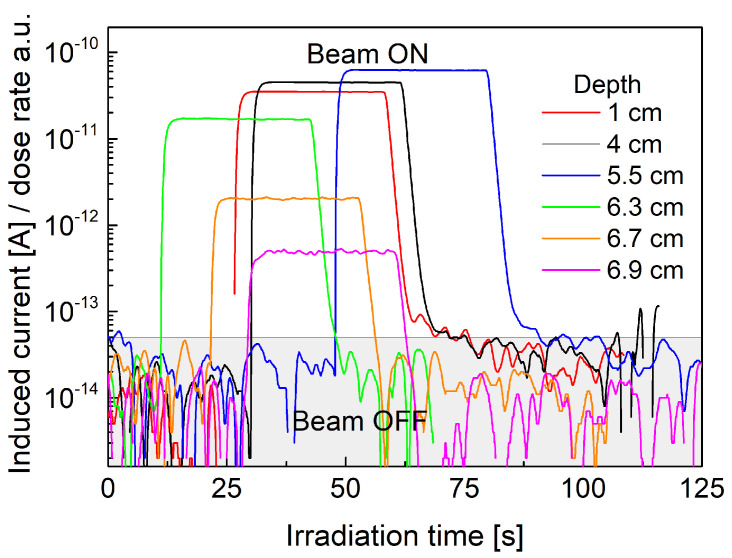
Proton beam-induced current in diamond GR microdosimeter at various depths of the plastic water phantom.

**Figure 5:**
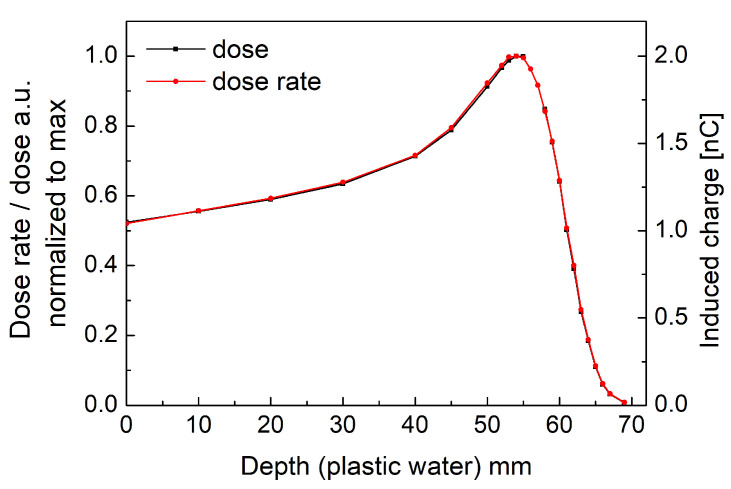
Comparison between measured induced current (dose rate profile) and measured induced charge (dose profile) for the 230 MeV proton beam degraded to 89 MeV.

Figure 4 presents beam-induced current curves measured under 30 s irradiations at various depths of plastic water phantom. The plateaus of the presented curves corresponds to relative dose rate of used proton beam. A remarkably low leakage current (less than $10^{-13}$ A) can be observed on the diamond sensor for beam-off conditions. The beam-induced charge was obtained by integrating the induced current over the irradiation window (the induced charge corresponds to relative absorbed dose). The maximum dose (induced charge) is obtained at 54 mm of plastic water depth marking the maximum peak of the depth dose profile. Comparison between dose rate, instantaneous induced current at the plateau level and dose integrated induced current curves (induced charge) within the irradiation time window is presented in Figure 5. It shows a perfect agreement between both quantities indicating the possibility of fast scanning in water phantoms if only constant beam current is guaranteed at the accelerator level.

To check reproducibility of the dose measurements, additional runs were performed for each depth of the depth dose profile. Three irradiations were performed at each depth and at the same irradiation times. Spread between measured values for each profile point was below 2%. This includes uncertainty of precision of dose delivery by accelerator. The result also shows the good quality of the underlying diamond material. Additionally, to our knowledge the presented sensor is the smallest volume $6.84\times 10^{-4}\;$ mm${}^{2}$ dosimeter available, which could be a perfect instrument for precise dose measurements in small-field, microbeams and mini beam dosimetry.

### 3.2. Energy Loss Spectra

**Figure 6:**
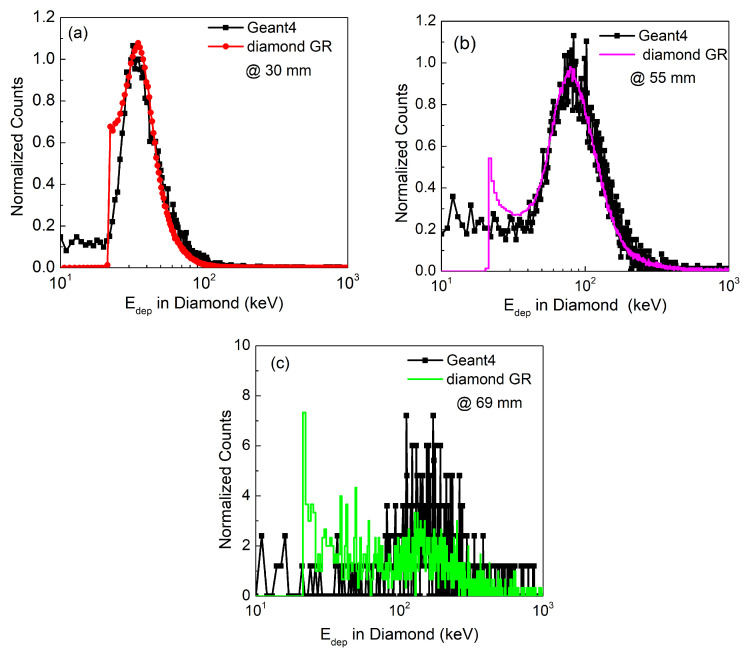
Selected calibrated pulse-height spectra obtained along depth dose profiles at (**a**) 30 mm, (**b**) 55 mm and (**c**) 69 mm. Counts have been normalized to the primary proton peak.

The energy loss spectra obtained from labZY MCA of the calibrated diamond GR microdosimeter is presented in Figure 6. Only three measurements, related to the characteristics points of the depth dose profile are shown; i.e., (a) at the plateau (30 mm), (b) at the Bragg peak (BP) maximum (55 mm depth) and (c) the distal end of the BP (69 mm).

The corresponding Geant4 simulated energy spectra at each characteristic point is included in Figure 6. There is a very good agreement between the measured spectra and the simulation result in terms of distribution shape, peak position and its width.

In addition, a progressive shift of the peak position of the energy loss spectra towards higher energies and broadening of the spectrum is observable with depth of water. This is due to protons being slowed down towards the BP. Also, there is an expected reduction in the intensity with depth. A low energy tail at around 22.25 keV is observed and become more pronounced at higher depth. This low energy tail was previously associated with the incomplete charge collection efficiency (CCE) due to the charge sharing between μSV and the guard ring electrode [21]. The measured spectra have a cut-off at 20 keV that corresponds to the current limit of the measurement set-up due to the electronic noise.

### 3.3. Water-Equivalent Microdosimetric Spectra

**Figure 7:**
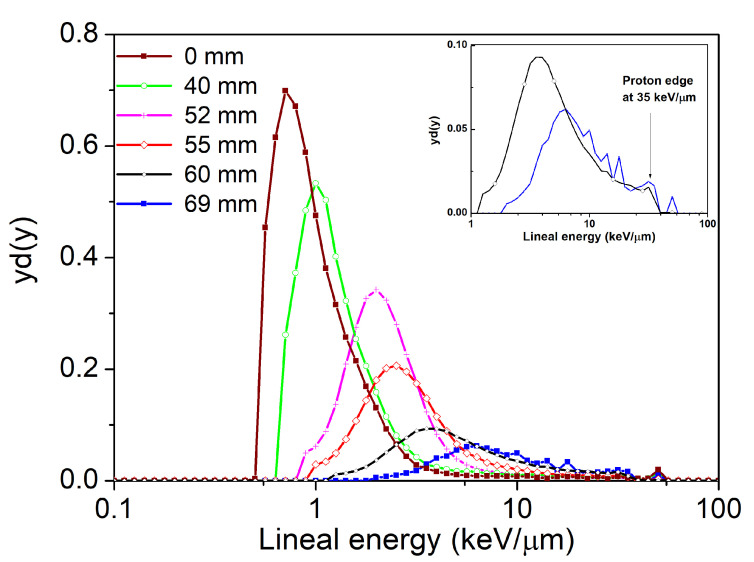
Experimental microdosimetric spectra obtained from a GR diamond microdosimeter in a plastic water phantom at depths of 0, 40, 52, 55, 60 and 69 mm. Inset: a zoom on the spectra at 60 and 69 mm. Proton edge at 35 keV/μm corresponds to protons of 1.3 MeV with a stopping range of approximately 12 μm in diamond equal to the sensor thickness.

**Figure 8:**
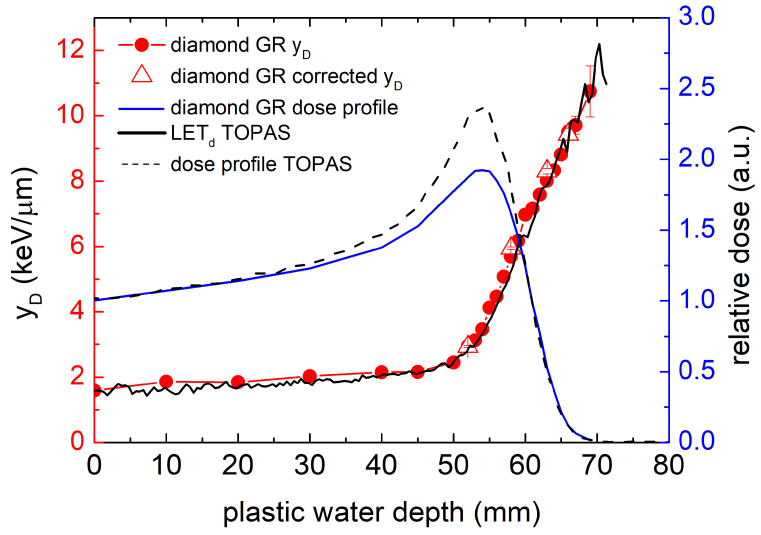
${\bar{y}}_{D}$ along the dose profile measured with a diamond GR microdosimeter compared with dose profile and LET${}_{d}$ from TOPAS simulations.

By using a diamond-water conversion factor of 0.32 [25], the measured energy deposition spectra obtained with the diamond GR microdosimeter was converted to water-equivalent microdosimetric spectra yd(y) as shown in Figure 7. In general, we observe that microdosimetric distributions shift towards higher energy values with increasing depth in plastic water phantom. Due to the nature of the spectra transformation process, the previously identified low energy tail in pulse-height spectra is still visible but does not significantly contribute to the microdosimetric spectra area and thus to the calculated ${\bar{y}}_{D}$ values. To confirm this, we performed a cut-off for four measured pulse height distributions removing low energy tails prior to the transformation to microdosimetric distributions. Four ${\bar{y}}_{D}$ values calculated from corrected microdosimetric spectra are displayed in Figure 8 with open triangles following the trend of ${\bar{y}}_{D}$ values obtained from not-corrected spectra. Furthermore, the typical “proton edge” can be seen from an enlarged view of the microdosimetric spectra measured at the BP and its distal part (see inset of Figure 7).

The maximum lineal energy observed at this edge was approximately 35 keV/μm in water. This lineal energy corresponds to a proton energy of 1.3 MeV with a stopping range of approximately 12 μm in diamond equalt to the sensor thickness. Therefore, this observed proton edge validates the energy calibration performed with the α-particle source and the pulse generator. Observed threshold in experimentally derived microdosimetric spectra amounts to 0.6 keV/μm water equivalent. This is comparable to frequently reported sensitivities of silicon based solid-state microdosimeters [26].

## 4. Discussion

### Comparison of Calculated ${\bar{y}}_{D}$ and Literature Results

The calculated dose-mean lineal energies ${\bar{y}}_{D}$ from experimentally measured microdosimetric spectra are presented in Figure 8. The microdosimetric spectra and ${\bar{y}}_{D}$ values measured with the diamond GR microdosimeter are in agreement with expected trends. The measured microdosimetric spectra shift to higher values of lineal energy with increasing penetration depth. The spectra shift, and thus, a steep increase of ${\bar{y}}_{D}$ is expected towards the end of the proton range, where the protons deposit more energy in μSVs. The obtained result is also consistent with microdosimetric spectral trends observed for silicon solid-state microdosimeters in clinical proton beams [27,28,29]. The dose-mean lineal energy ${\bar{y}}_{D}$ values in the entrance region of the BP (0 mm to 40 mm) were approximately 2 keV/μm. Identical values were reported for SOI microdosimeter for 159 MeV proton pencil beam [27]. In the proximal and distal parts of the BP (52 mm to 69 mm), the measured values went from 3 to 10.7 keV/μm, and the maximum measured ${\bar{y}}_{D}$ value was reached at very distal part of BP at 69 mm depth at the point of only 1% relative dose distribution. Similar values were reported for SOI microdosimeter ranging from 3 to 10.3 keV/μm, with the maximum ${\bar{y}}_{D}$ value reached at 20% of relative dose distribution in BP distal fall-off. It must be noted that if we compare the dose profile at the BP distal fall-off and ${\bar{y}}_{D}$ values, in general, diamond measured ${\bar{y}}_{D}$ values for a degraded 230 MeV proton beam are lower compared to reported ${\bar{y}}_{D}$ values for SOI microdosimeter measured for lower proton beam energies. For instance, if we consider the measured value of ${\bar{y}}_{D}$ = 5.3 keV/μm in diamond, it corresponds to a position at 50% of relative dose in the BP distal fall-off part of a degraded 230 MeV proton beam. For the SOI microdosimeter, the ${\bar{y}}_{D}$ at 50% of the relative dose in the BP distal fall-off part is 8.4 keV. Again, such a trend is expected, since with the increase of the initial beam energy, the maximum ${\bar{y}}_{D}$ decreases. The longer traveling path from a higher-energy beam can result in more energy straggling close to the maximum range at the corresponding locations. This will result in a higher mean energy and a lower ${\bar{y}}_{D}$, accordingly [30].

To further benchmark our experimental results of ${\bar{y}}_{D}$, we performed numerical simulations using TOPAS Simulation Toolkit [31] to obtain dose-weighted LET${}_{d}$ values. The complete Y1 beam line geometry (Figure 3) with a configuration identical to the experiment was implemented in the simulation. We used a water phantom placed in the isocenter to obtain dose and LET${}_{d}$ profiles. The TOPAS ProtonLET scorer gives the LET${}_{d}$ of primary and secondary protons, including the energy deposited by associated secondary electrons. More details about LET${}_{d}$ scoring technique in TOPAS/Geant4 can be found in [32,33]. The TOPAS-simulated LET${}_{d}$ profile is displayed in Figure 8 (in black solid line); it is in close agreement with the experimentally measured ${\bar{y}}_{D}$ profile for all depths. Frequently, due to the fundamental difference between LET${}_{d}$ and ${\bar{y}}_{D}$, literature-reported comparisons between both differ, especially in the entrance part of the BP [28]. In general, this difference arises from the different volumes (voxels) used for LET${}_{d}$ calculation (typically ≈ 1 mm) and ${\bar{y}}_{D}$ measurement (typically ≈ dozens of microns) and is related to the energy transfer differences by the delta-rays. Both quantities frequently differ at entrance part of the BP where delta-rays have higher range, exceeding the volume of interest used in measurement. In the distal part, the delta-rays’ range becomes comparable with the volume of interest used in measurement; thus, both values are in close agreement. In our case, volumes in simulation (0.05 mm) and measurement (≈39 microns water-equivalent) were comparable, resulting in close agreement of ${\bar{y}}_{D}$ and LET${}_{d}$ in the whole range. A comprehensive review of volume size influence on LET${}_{d}$ calculations can be found in [30].

The TOPAS-simulated dose profile (shown in dash line of Figure 8) reproduces very well the distal part of the measured diamond GR microdosimeter dose profile (shown in solid blue line) and the position of the BP. However, its amplitude differs from the measured one. Both profiles have been normalized to the entrance point. At this stage of sensors’ development, we believe that the observed difference arises from characteristics of the diamond sensor, most probably due to the charge collection inefficiency or induced proton beam DC current saturation in the peak region for the measurement. Our second hypothesis (less probable) assumes that this difference arises from the over-response of the sensor in the entrance part due to the presence of non-tissue equivalent materials around the diamond sensor. Further experiments with reference ionization chambers are planned to clarify this issue. Nevertheless, the measured dose profile already allows precise sensor positioning and proton range verification.

## 5. Conclusions and Current Perspectives

A diamond GR microdosimeter with an energy-calibrated response has been fabricated and tested in a proton therapy facility. A demonstration of simultaneous measurement of the dose depth profile and microdosimetry spectra has been reported. The possibility of measuring both the depth dose profile and pulse-height spectra using one sensor allows precise positioning of the device for microdosimetric spectra acquisition at the selected points-of-interest—a very useful feature for a future beam quality assurance system in hadron therapy. The calibrated pulse-height spectra of the energy loss of protons in diamond have been benchmarked with a Geant4 simulation, showing very good agreement in terms of maximum peak positions and widths. The dose-mean lineal energy ${\bar{y}}_{D}$ values have been compared and discussed with the experimental data measured with silicon based solid-state microdosimeters, as reported in the literature, and with numerical calculations of LET${}_{d}$ values. We saw all these values to be in close agreement at the entrance, and at the proximal and distal parts of the BP. Finally, we reported a very good correlation between TOPAS-simulated dose profile and our measurement for the distal part of the BP. A difference in dose profile amplitude at the BP could be associated with the charge collection characteristics of the diamond sensor.

In ongoing work, the system is being miniaturized by integrating the CSA on the same printed circuit board as the sensor, and the modular elements can be integrated into one water-proof PMMA housing to assure full portability. Thus, the entire system is expected to be compact, to be battery powered and to have wireless data transfer capability. In order to improve the quality of the pulse height spectra, a new type of diamond membrane microdosimeter with truly isolated 3D microSVs, surrounded by a tissue equivalent, non-electrically active material is envisaged for future developments of the microdosimetry system.

## 6. Patents

Patent pending: System for Dosimetric and Microdosimetric Ionizing Radiation Charcaterization—I.A. Zahrdnik, M. Pomorski, EP20305733.6, 30 June 2020.

## Data Availability

Data reported in this article are available on request from the corresponding authors.
